# Clinical Outcomes of Hidden Lesions of the Medial Meniscus Treated With a Flexible All-Inside Device

**DOI:** 10.7759/cureus.109046

**Published:** 2026-05-17

**Authors:** Kensuke Tanaka, Kimihiko Makiyama, Yoshinori Tojo, Yoshiyuki Hara, Kazuto Hirai, Ryosuke Kajimoto, Kyosuke Hibi, Toshinori Tamada, Kenjiro Hasegawa, Tomoyuki Noda

**Affiliations:** 1 Department of Orthopaedic Trauma Surgery and Sports Medicine, Kawasaki Medical School General Medical Center, Okayama, JPN

**Keywords:** anterior cruciate ligament reconstruction, flexible all-inside device, meniscal repair, ramp lesion, second-look arthroscopy

## Abstract

Purpose

Ramp lesions are characteristic injuries associated with anterior cruciate ligament (ACL) rupture and involve disruption of the meniscus-synovium interface near the posterior horn of the medial meniscus. Among these, tibiofibular side-only tears at the bursal attachment, so-called hidden lesions, are difficult to visualize from the standard anterior portal, making their diagnosis and treatment challenging. This study examined the outcomes of suturing hidden lesions using an all-inside technique with the FAST-FIX device from the anterior portal, with a focus on clinical results and the healing status assessed by postoperative second-look arthroscopy.

Methods

Forty patients with hidden lesions repaired using the FAST-FIX device (Smith & Nephew, Hertfordshire, UK) who underwent second-look arthroscopy from 2020 to 2023 were included. Until 2022, a non-flexible device (FAST-FIX 360), in which the shaft does not bend, was used (n = 21). After 2022, a flexible device (FAST-FIX FLEX), which allows bending of the needle and shaft, was used (n = 19). Meniscal healing was evaluated during second-look arthroscopy, and clinical outcomes were assessed using the Lysholm score and Knee Injury and Osteoarthritis Outcome Score (KOOS).

Results

With the non-flexible FAST-FIX 360, complete healing, partial healing, and re-tear were observed in 62%, 9%, and 29% of cases, respectively. In contrast, with the flexible FAST-FIX FLEX, complete healing, partial healing, and re-tear were observed in 74%, 21%, and 5% of cases, respectively. The flexible device demonstrated a significantly lower re-tear rate than the non-flexible device. Although the complete healing rate was higher in the flexible group, the difference was not statistically significant. Lysholm scores and KOOS did not differ significantly between the two groups.

Discussion

The anterior-portal repair of ramp lesions using all-inside devices results in modest healing rates due to the difficulty of penetrating the posterior horn. In the present study, incomplete healing occurred in 29% of cases using the non-flexible device, whereas the failure of healing with the flexible device was observed in only 1 of 19 cases. The flexibility of the device allows for better capture of the posteroinferior capsule and meniscotibial ligaments, facilitating a more secure repair. Therefore, the anterior-portal repair of hidden ramp lesions using a flexible all-inside device may achieve favorable healing outcomes.

## Introduction

A ramp lesion is a characteristic injury associated with anterior cruciate ligament (ACL) rupture and is defined as a disruption of the meniscus-synovial interface near the posterior horn of the medial meniscus (MM) [1]. Ramp lesions have been reported in approximately 20-40% of patients with ACL injuries [2,3]. When an ACL injury is accompanied by a ramp lesion, anterior tibial translation and external rotation are further increased, and ACL reconstruction alone may not adequately restore knee stability [4]. In addition, untreated ramp lesions may enlarge over time and lead to locking symptoms of the MM [5]. However, ramp lesions are frequently overlooked during a routine arthroscopic evaluation using the standard anterior portal [6].

Among ramp lesions, tears that occur only on the tibial side at the meniscocapsular attachment are referred to as hidden lesions because they are difficult to detect from the standard anteromedial, anterolateral, or posterolateral portal. Hidden lesions are prone to being underdiagnosed due to their limited visualization [7]. Although some stable lesions may heal without repair, unstable lesions are generally recommended for surgical repair to restore knee stability [8].

Furthermore, limited evidence is currently available on optimal repair techniques for hidden lesions, particularly when using all-inside devices from the anterior portal. Therefore, we herein describe the repair of hidden ramp lesions using an all-inside FAST-FIX device (Smith & Nephew, Hertfordshire, UK) from the anterior portal and evaluated clinical outcomes, including the healing status confirmed by second-look arthroscopy.

## Materials and methods

Subjects and methods

This study was conducted at Kawasaki Medical School General Medical Center in Okayama Prefecture, Japan. Patients were consecutively enrolled between January 2020 and December 2023. No formal sample size calculation was performed; the sample size was selected based on the number of eligible patients treated during the study period. A total of 120 patients who underwent arthroscopic ACL reconstruction during the study period were included.

**Figure 1:**
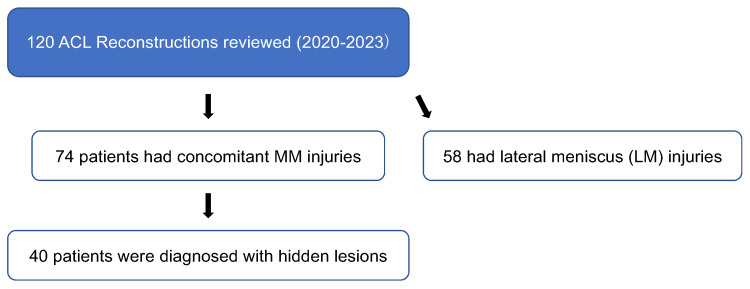
Patient selection flowchart Flowchart illustrating the patient selection process. Of 120 patients who underwent anterior cruciate ligament (ACL) reconstruction, 40 patients with hidden lesions were included in the final analysis. MM: medial meniscus The figure was created using Microsoft PowerPoint (Microsoft Corporation, Redmond, WA, US).

Inclusion criteria were patients who underwent arthroscopic ACL reconstruction with concomitant meniscal injury at our institution. There were no specific exclusion criteria, except for patients with incomplete clinical data or insufficient follow-up. Data were collected through a retrospective review of consecutive cases to minimize any selection bias and reflect real-world clinical practice. The patient selection process is summarized in Figure 1.

**Figure 2:**
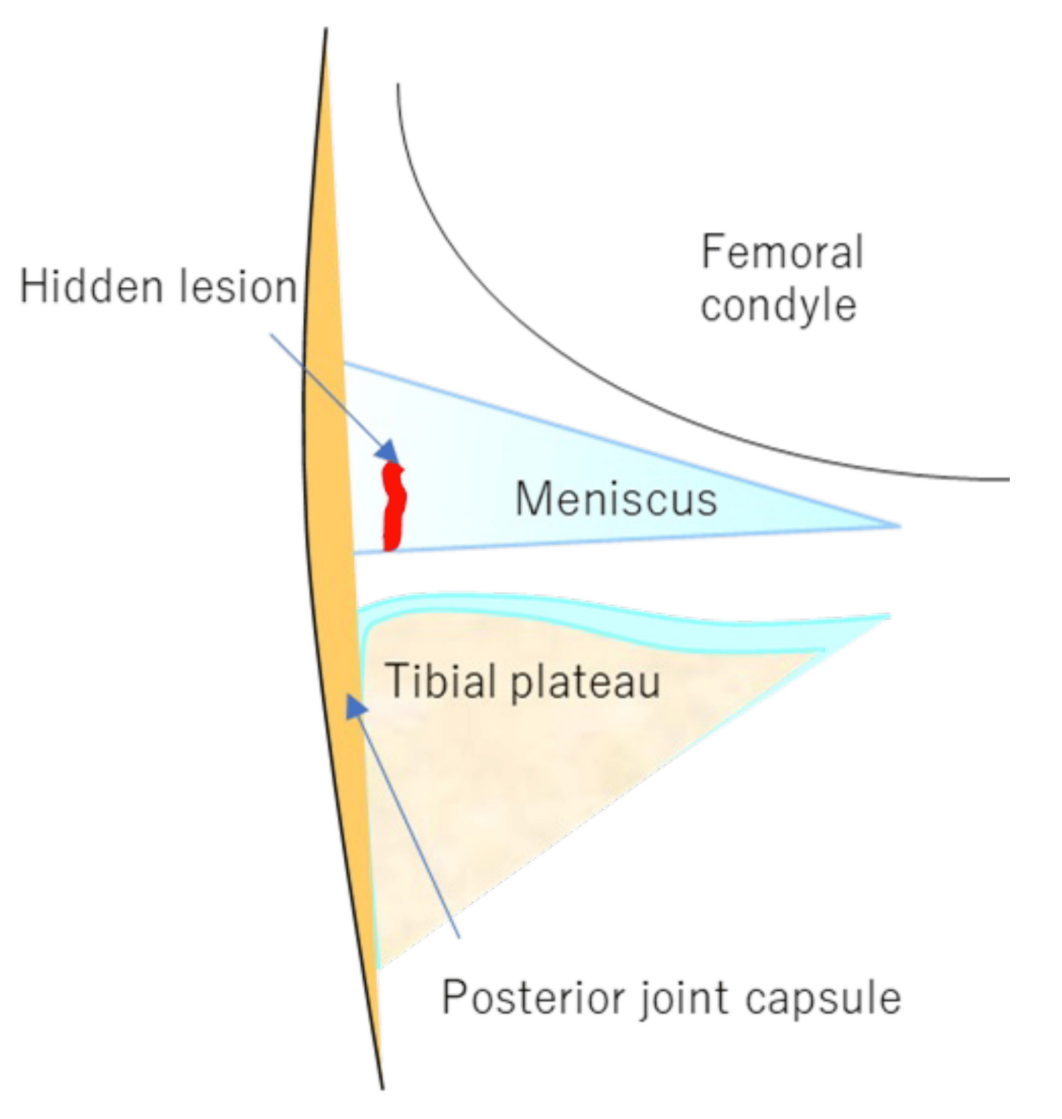
Schematic illustration of a type 3 ‘hidden’ ramp lesion Hidden lesions are defined as tibial-side tears at the meniscocapsular attachment, corresponding to type 3 in the ramp lesion classification described by Thaunat et al. [7]. This image was created using Microsoft PowerPoint (Microsoft Corporation, Redmond, WA, US).

Of the 120 patients who underwent ACL reconstruction, 74 had concomitant MM injuries, and 58 had lateral meniscus (LM) injuries; some patients had both injuries. Among the patients with MM injuries, 40 met the arthroscopic diagnostic criteria for hidden lesions and were included in the final analysis. Hidden lesions were defined as tibial-side tears at the meniscocapsular attachment, corresponding to type 3 of the ramp lesion classification described by Thaunat et al. (Figure 2) [7].

**Figure 3:**
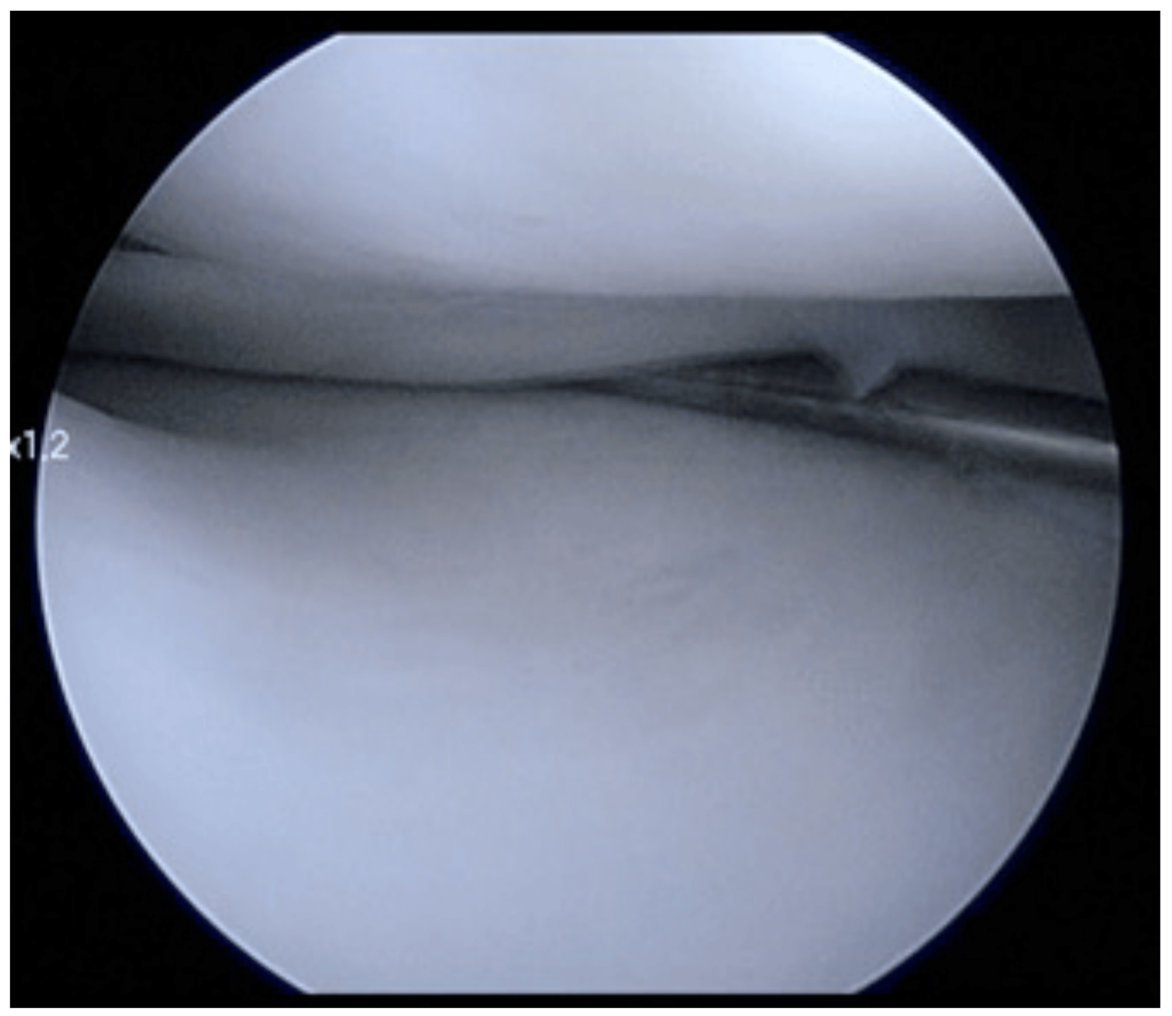
Arthroscopic assessment of medial meniscus instability Arthroscopic image demonstrating the instability of the posterior horn of the medial meniscus when pulled anteriorly with a probe. The image was obtained at our institution.

Hidden lesions were diagnosed through anterior-portal observations using the following criteria: (1) an elevation of the posterior horn of the MM, allowing clear visualization of the posterior capsule; (2) instability observed when the posterior horn of the MM is pulled anteriorly with a probe (Figure 3); and (3) no tear identified on the superior aspect of the meniscocapsular junction from the posterior angle to the posterior segment in the notch view.

Treatment was performed using an all-inside device introduced through the anterior and posterior portals. Vertical sutures were placed across the inferior aspect of the tear, with one to three stitches being applied depending on the tear size.

ACL reconstruction was simultaneously performed. Anatomic double-bundle reconstruction using an autologous hamstring tendon was conducted on 33 patients, a bone-patellar tendon autograft on 6, and a quadriceps tendon autograft on 1. The mean age of patients was 25.8 years (range, 14-50 years), and the cohort included 18 males and 22 females.

The choice of the all-inside device was based on the institutional standard treatment protocol, which was updated in 2022 from the use of a non-flexible device (FAST-FIX 360) to a flexible device (FAST-FIX FLEX), rather than being selected using a prospective comparative study design. The all-inside devices used were the FAST-FIX 360 and FAST-FIX FLEX.

**Figure 4:**
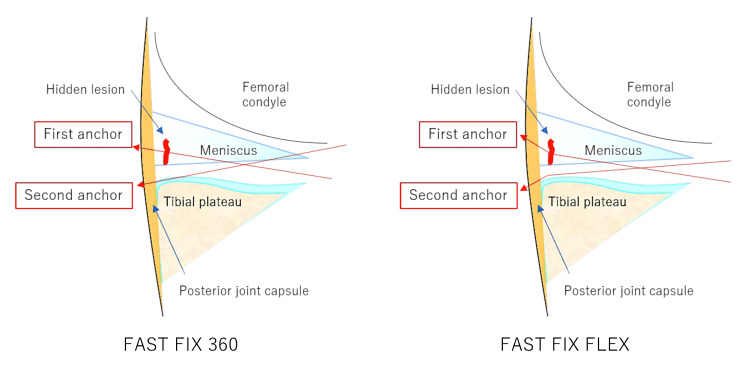
Insertion trajectories of the FAST-FIX 360 and FAST-FIX FLEX devices This image was created using Microsoft PowerPoint (Microsoft Corporation, Redmond, WA, US).

The FAST-FIX 360 is a rigid device that cannot be used in a bent position, whereas the FAST-FIX FLEX is designed to allow flexible, bent insertion (Figure 4).

The meniscal healing status at follow-up was evaluated using the classification described by Seo et al. [9]. Clinical outcomes were assessed using the Lysholm score [10] and the Knee Injury and Osteoarthritis Outcome Score (KOOS) [11]. The primary endpoint was arthroscopic healing status assessed at second-look arthroscopy. Statistical analyses were performed using StatMate V (version 5.01, GraphPad Software, San Diego, CA, USA). The Mann-Whitney U test and χ2 test were used, with the significance level set at p < 0.05.

Ethical consideration

The present study was conducted in accordance with the ethical principles of the Declaration of Helsinki, and was approved by the Ethics Committee of Kawasaki Medical School and Hospital (Approval No.7043-000). Written informed consent was waived due to the retrospective design, and an opt-out approach was used by publicly disclosing study information.

## Results

No significant differences were observed in age, sex, or the side of injury between the two groups. Postoperative re-examinations showed negative results in both the Lachman and pivot-shift tests, with no evidence of instability in either group. The mean side-to-side difference in anterior tibial translation was 0.38 mm in the FAST-FIX FLEX group and 0.47 mm in the FAST-FIX 360 group, with no significant between-group difference.

Second-look arthroscopy revealed complete healing in 13 of 21 knees (62%), partial healing in 2 (9%), and re-tear in 6 (29%) in the FAST-FIX 360 cohort. In the FAST-FIX FLEX cohort, complete healing was observed in 14 of 19 knees (74%), partial healing in 4 (21%), and re-tear in 1 (5%) (Table 1). Fisher’s exact test demonstrated a significantly lower re-tear rate in the FAST-FIX FLEX group than in the FAST-FIX 360 group (5% vs 29%, p = 0.046; risk difference, 24%; 95% CI, 3%-45%). In contrast, although the complete healing rate was higher in the FAST-FIX FLEX group (74% vs 62%), the difference was not statistically significant (p = 0.49; risk difference, 12%; 95% CI, −17% to 41%).

**Table 1:** Degree of meniscal healing at second-look arthroscopy Data are presented as number (percentage). The chi-square (χ²) test was used for statistical analysis, with p < 0.05 considered statistically significant.

	FAST-FIX 360 (n = 21)	FAST-FIX FLEX (n = 19)
Complete healing	13 (62%)	14 (74%)
Partial healing	2 (9%)	4 (21%)
Re-tear	6 (29%)	1 (5%)

**Figure 5:**
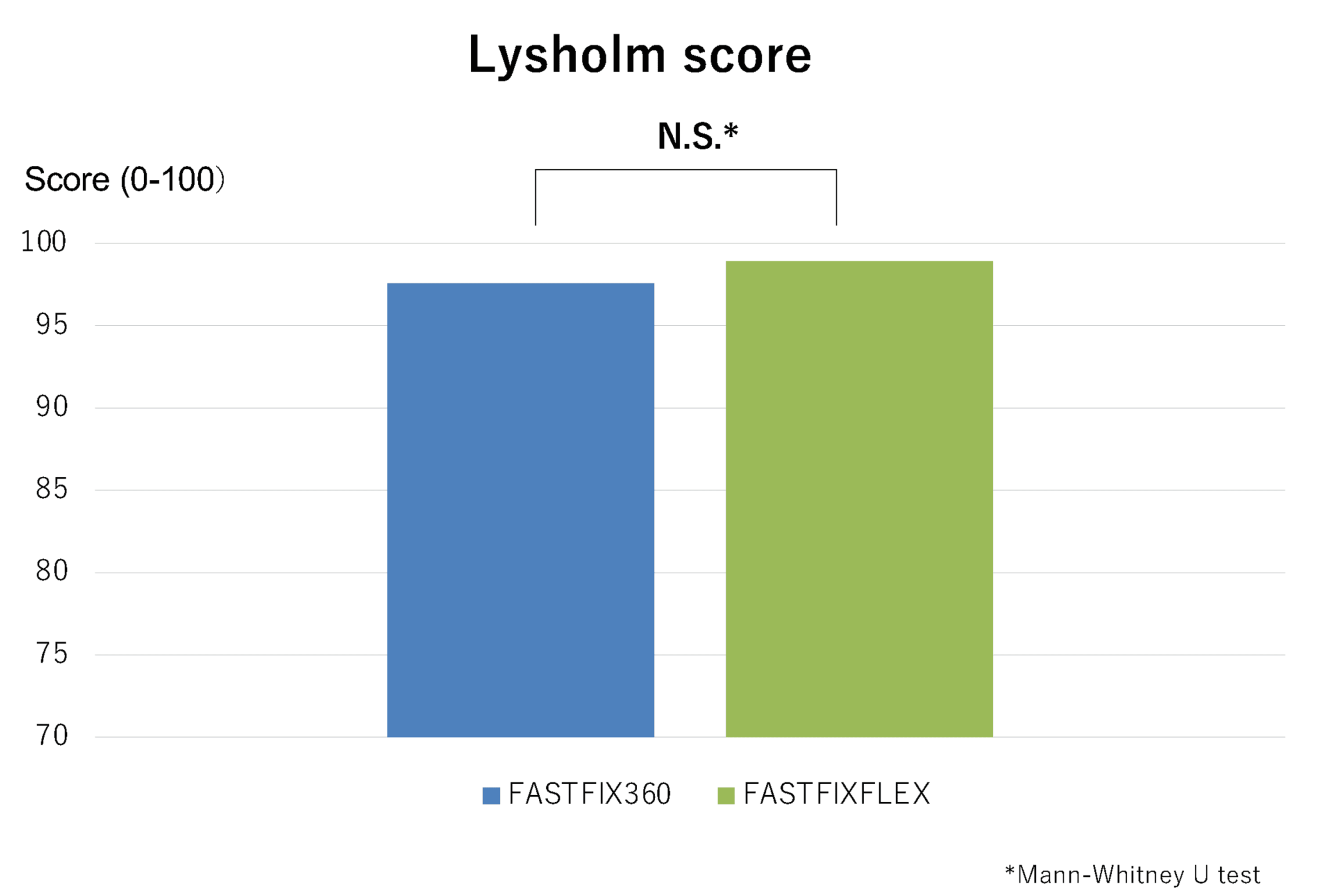
Comparison of Lysholm scores between the two groups The mean Lysholm score did not differ significantly between the groups. Error bars indicate standard deviations. Statistical analysis was performed using the Mann–Whitney U test. N.S., not significant

**Figure 6:**
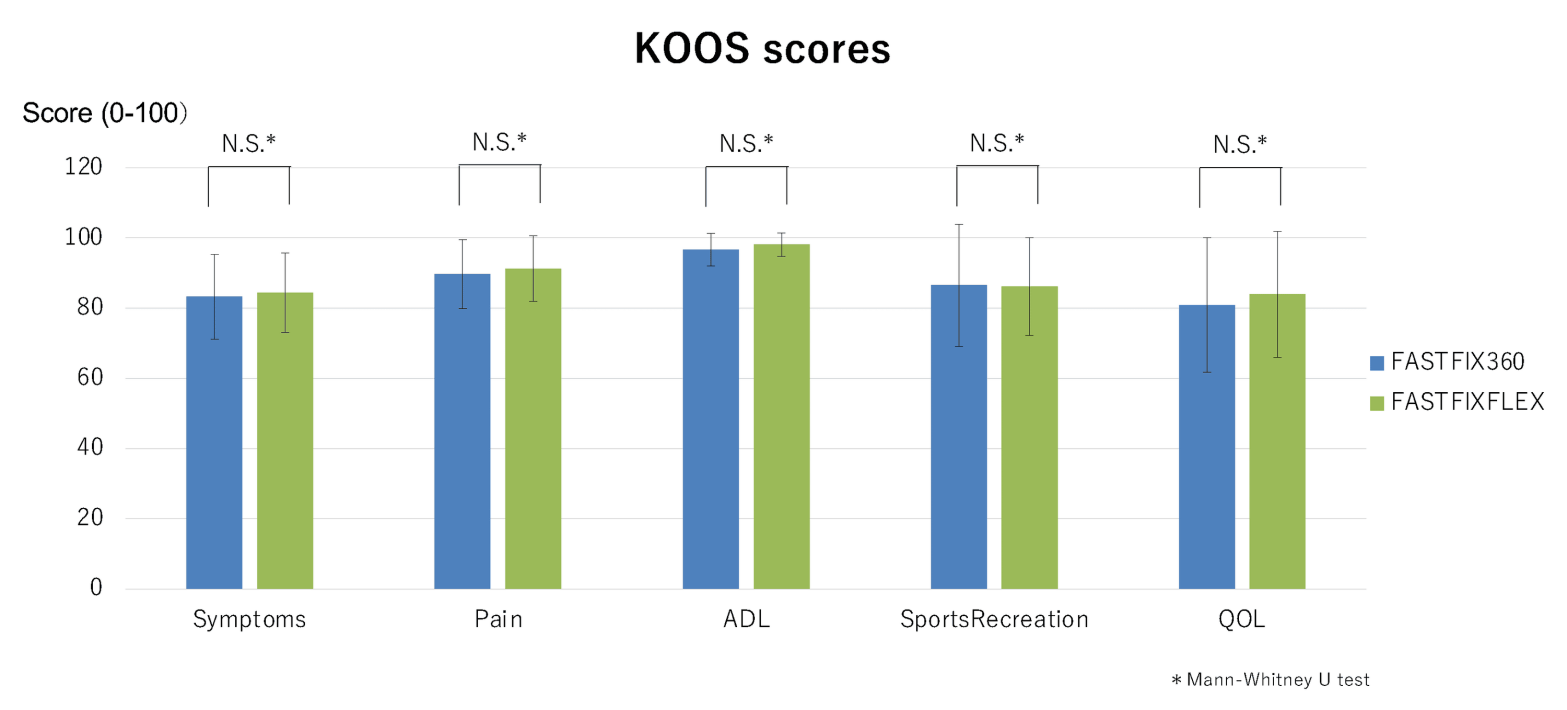
Comparison of KOOS scores between the two groups No significant differences were observed in any of the five KOOS scores (Symptoms, Pain, Activities of Daily Living (ADL), Sports and Recreation, and Quality of Life (QOL)). Error bars indicate standard deviations. Statistical analysis was performed using the Mann–Whitney U test. KOOS, Knee injury and Osteoarthritis Outcome Score; N.S., not significant

Clinical outcomes did not significantly differ between the two cohorts. Mean Lysholm scores were 97.3 in the FAST-FIX 360 group and 98.1 in the FAST-FIX FLEX group. Similarly, no significant differences were observed in any of the five subscales of KOOS (Figure 5) (Figure 6).

## Discussion

Ramp lesions have not yet been clearly defined or consistently diagnosed. Thaunat et al. classified these lesions into five types based on the morphology and location of arthroscopic findings [7]. Type 1 involves damage to the superficial meniscocapsular ligament; type 2 is a meniscal tear at the femoral attachment; type 3 is a tear on the tibial side at the capsular attachment; type 4 represents a longitudinal meniscal tear at the capsular attachment; and type 5 is a combination of a longitudinal meniscal tear at the capsular attachment and an anterior longitudinal tear.

In the narrow sense, ramp lesions are regarded as insufficiency tears of the meniscus or meniscotibial ligament at the tibial attachment. In the present study, we focused on type 3 lesions, which are difficult to detect using conventional arthroscopy and, thus, are described as hidden lesions.

A diagnosis was made by observing the posteromedial meniscus through the anterior portal, with clear visualization of the posterior capsule. Probing enabled anterior displacement of the posterior MM, revealing instability. Cases in which no tear was visible from the superior aspect under the notch view were classified as type 3 hidden lesions and treated with suturing of only the inferior aspect.

A biomechanical study previously demonstrated that meniscocapsular and meniscotibial lesions significantly increased knee laxity in ACL-deficient knees, and their repair contributed to the restoration of joint stability [3]. These findings support the clinical importance of accurately identifying and repairing hidden lesions.

The reported incidence of ramp lesions associated with ACL injuries ranges from 9% to 31% [12], partly due to inconsistencies in definitions and diagnostic techniques. Ramp lesions, particularly hidden lesions, are frequently overlooked during a routine arthroscopic evaluation when only the standard anterior portal is used [6,9]. This diagnostic difficulty may contribute to variability in the reported incidence of ramp lesions and highlights the importance of careful probing and visualization of the posteromedial compartment.

The indication for repair of ramp lesions remains controversial. Previous studies, including a randomized controlled trial by Liu et al., have suggested that stable ramp lesions may not require repair, as no significant differences in clinical outcomes were observed between the repair and non-repair groups [8]. In the present study, however, even lesions with mild instability were treated surgically, which may have influenced the observed incidence and outcomes. Therefore, careful assessment of lesion stability may be important in determining the optimal treatment strategy.

Regarding hidden lesions, Sonnery-Cottet et al. reported a rate of 16.8% [2]. In our series, 40 of 120 cases (33%) had hidden lesions, which is slightly higher than the previously reported rate. Although some studies indicated that stable ramp lesions may heal without repair, increasing consensus suggests that unstable lesions need to be repaired in order to prevent persistent instability and the potential progression of meniscal damage [8]. In the present study, even slight instability detected by probing the inferior aspect of the posterior MM was diagnosed as a hidden lesion and treated by suturing, which may explain the higher incidence.

The all-inside device FAST-FIX 360 places two implant anchors across the tear. The device uses a trigger mechanism to draw the meniscus toward the joint capsule and secure the anchors. FAST-FIX 360 is widely used due to its high fixation strength and suitability for mid-to-posterior meniscal tears. Depending on the tear morphology, it allows vertical, horizontal, and tie-grip suturing techniques [13].

However, several limitations have been noted. Since the implant is delivered through a rigid shaft, the same portion of the shaft must be advanced during insertion. This design may restrict access to medial posterior segment tears, such as ramp lesions, and hinder full penetration of the posterior capsule due to interference from the medial femoral condyle. As a result, several studies have documented suture failure [14]. For example, Ito et al. reported a complete healing rate of only 46% for ramp lesions repaired from the anterior portal using FAST-FIX 360 [15].

To overcome these issues, some techniques involve creating a posteromedial portal and using a suture-hook device [5], or inserting the all-inside device from the anterior portal while elevating the meniscotibial ligament with a grasper introduced through the posteromedial portal. Another implant anchor may then be placed at the outer one-third of the meniscus to facilitate fixation [16]. However, these approaches are technically demanding and require advanced arthroscopic skills, which may limit their widespread use [16].

FAST-FIX FLEX, introduced in 2022, is a flexible all-inside device in which both the needle and shaft may be bent using an attached instrument. In contrast to rigid, straight-shaft devices, which may impinge on the medial femoral condyle and hinder access to the intended repair site, the flexible shaft may be curved along the contour of the medial femoral condyle, enabling safer and more secure access to the posteroinferior capsule while minimizing the risk of iatrogenic cartilage injury. This flexibility enables insertion while avoiding the medial femoral condyle, thereby broadening the range of applicable tear locations. In the present study, approximately 30% of hidden lesions re-tore when repaired with the non-flexible FAST-FIX 360 through an anterior portal, whereas only 1 of 19 cases re-tore when repaired with FAST-FIX FLEX. The ability to adjust the trajectory of the flexible device facilitates access to the posterior segment of the MM and allows the secure insertion of anchors into the inferior surface of the posterior capsule and meniscus. These results suggest that improved structural healing may be achievable even when such lesions are repaired from the anterior portal using an all-inside technique. However, although improved healing rates were observed, no significant differences were found in clinical outcomes. This discrepancy may be explained by a ceiling effect, as postoperative Lysholm and KOOS scores were already high in both groups, thereby limiting the ability to detect differences. In addition, the relatively short follow-up period may have contributed to the absence of observable clinical differences.

Limitations

Several limitations should be acknowledged. First, as this was a single-center retrospective study, the findings may have limited generalizability. Second, there is a potential temporal bias related to the chronological change in devices. Because the flexible device was introduced later, improvements in surgical experience and technique over time may have influenced the results, thereby potentially affecting the internal validity of the study. Third, meniscal healing was assessed by a single observer, which may have introduced an observer bias. Fourth, the follow-up period was relatively short, with a minimum of one year, and may not have been sufficient to fully evaluate long-term clinical outcomes and the healing status. Therefore, further studies with larger sample sizes, multicenter designs, and longer follow-up periods are warranted.

## Conclusions

The present study examined the outcomes of hidden lesions repaired using an all-inside device. Hidden lesions were identified in 33% of patients with ACL injuries. A high re-tear rate was observed with non-flexible all-inside devices, whereas the use of a flexible device may be associated with improved healing outcomes. Future studies need to include multicenter prospective designs with larger sample sizes and longer follow-up periods to more accurately evaluate long-term clinical outcomes and the healing status of hidden lesions. Direct comparative studies between flexible and non-flexible devices are also warranted.
